# The experiences of critical care nurses caring for patients undergoing awake prone positioning: a qualitative study

**DOI:** 10.1186/s12912-026-04507-0

**Published:** 2026-03-11

**Authors:** Hanyang Su, Tianji Zhou, Xiaoning Yu, Sihe Deng, Weihong Wang, Jingmei Lu

**Affiliations:** 1 Teaching and Research Section of Clinical Nursing, Xiangya Hospital, Central South University, Changsha, China; 2 Department of Respiratory Medicine, National Key Clinical Specialty, Branch of National Clinical Research Center for Respiratory Disease, Xiangya Hospital, Central South University, Changsha, China; 3 Medical College of Hunan Normal University, Changsha, China; 4 National Clinical Research Center for Geriatric Disorders, Xiangya Hospital, Central South University, Changsha, China; 5 Xiangya School of Nursing, Central South University, Changsha, China

**Keywords:** Awake prone positioning, Critical care nurses, Qualitative research, Nurses’ experiences, Psychological support

## Abstract

**Background:**

Awake prone positioning is increasingly used for patients with respiratory distress, and understanding the experiences of critical care nurses can illuminate insights into best practices, emotional and physical impacts on the caregivers, and potential areas for support and training improvements. This qualitative study aims to fill a gap in existing research by providing a deeper understanding of the nursing care involved in awake prone positioning from the frontline caregivers’ viewpoint.

**Methods:**

A descriptive qualitative research design was employed to capture the rich and nuanced experiences of critical care nurses. Sixteen participants were purposively selected from seven intensive care units (ICUs) across four tertiary hospitals where awake prone positioning was routinely implemented. Semi-structured interviews were conducted. Content analysis was applied to extract key themes from the interview data.

**Results:**

This study generated six main themes: (1) The role of nurse in APP Implementation, (2) Technical challenges in APP, (3) Team collaboration and communication, (4) Provision of patient psychological support, (5) Training and educational needs, and (6) Nurses’ Burden. These findings revealed that critical care nurses play a crucial role in the successful implementation of awake prone positioning, encountering significant technical challenges and requiring effective team collaboration and communication to ensure optimal patient care. Critical care nurses also provided psychological support to patients, addressing both emotional and physical responses to the procedure. Additionally, the findings highlighted the importance of ongoing training and education for critical care nurses to handle the demands of awake prone positioning, as well as the physical and emotional burdens faced by critical care nurses in this high-stress environment.

**Conclusion:**

This study sheds light on the intricate experiences of critical care nurses in awake prone positioning. By recognizing and addressing these aspects, we will contribute to an environment that not only optimizes patient care but also nurtures the well-being and resilience of the nursing workforce. Future research should further explore sociocultural influences and patient perspectives to refine interventions and elevate the standards of care in awake prone positioning.

**Clinical trial number:**

Not applicable.

**Supplementary Information:**

The online version contains supplementary material available at 10.1186/s12912-026-04507-0.

## Background

 In critical care settings, the delivery of optimal care to patients undergoing unique and advanced ventilation techniques is paramount. One such technique gaining prominence is awake prone positioning (APP), a method employed to improve oxygenation and ventilation in patients [1–3], particularly those with conditions such as acute respiratory distress syndrome (ARDS) [4, 5]. In this method, patients are positioned on their stomachs (prone position) while remaining conscious and alert (awake) [6]. Unlike traditional prone positioning, which may involve sedation or unconsciousness [7, 8], the awake approach allows patients to actively participate in their care. This active participation potentially contributes to better outcomes and reduces the need for sedative medications [9].

As this approach becomes more integrated into critical care practices [10], it is essential to explore and understand the experiences of healthcare professionals, specifically critical care nurses, who play a central role in the implementation and management of awake prone positioning [11, 12]. Unlike traditional ventilation techniques, APP presents unique technical challenges, such as the need for precise positioning and ongoing monitoring while the patient is awake, which requires constant adjustments. Emotionally, critical care nurses must provide continuous psychological support, as patients are conscious and may experience anxiety or discomfort during the procedure. These distinct technical and emotional demands underscore the importance of qualitative exploration to capture the nuanced experiences and challenges faced by nurses in managing APP, which are not typically encountered with other ventilation methods [13].

Critical care nurses play a central role in managing awake prone positioning, which involves ensuring patient safety, monitoring vital signs, and providing emotional support [13]. These tasks require both technical precision and psychological care, which are crucial to the overall success of the intervention. This study aims to explore these unique nursing experiences and challenges in order to improve understanding and support for nurses working with awake prone positioning.

Current research on awake prone positioning mainly focuses on its efficacy, safety, and the practical aspects of its implementation in non-intubated patients, particularly those with COVID-19-induced acute hypoxemic respiratory failure [14]. Studies explore how awake prone positioning can improve oxygenation and potentially reduce the need for intubation. However, there is a scarcity of qualitative research exploring nurses’ feelings, challenges, and coping strategies while caring for patients in awake prone positioning.

To address this gap, the following research questions were developed to guide the study: What are the experiences and challenges faced by critical care nurses caring for patients undergoing APP? How do these experiences inform improvements in nursing practice, education, and institutional policies related to APP? This study aims to provide in-depth insights into the multifaceted aspects of nursing care in APP, offering a qualitative exploration that goes beyond quantitative metrics.

This study is necessary as it provides in-depth insights into the experiences and challenges faced by critical care nurses caring for patients undergoing awake prone positioning, a practice that has gained prominence for managing certain respiratory conditions. The utilization of awake prone positioning represents a paradigm shift in respiratory care, requiring not only technical proficiency but also a deep understanding of patient responses and individualized nursing interventions [15]. As such, this study endeavors to shed light on the multifaceted aspects of nursing care in this context, offering a qualitative exploration that goes beyond quantitative metrics. Through this research, we aspire to provide in-depth insights into the experiences of critical care nurses in awake prone positioning and to identify areas for potential improvement in training, support, and overall patient outcomes.

## Methods

### Study design and setting

This study employs a descriptive qualitative research design to explore the experiences of critical care nurses caring for patients undergoing awake prone positioning. This approach is chosen to capture the complex, nuanced perspectives and challenges faced by nurses in this specific clinical context. The research is conducted in seven intensive care units (ICUs) across four urban, tertiary hospitals in Hunan Province, China. These hospitals are classified as Grade A tertiary hospitals, indicating they are at the highest level of hospitals in China in terms of their capacity to provide comprehensive medical care, perform advanced medical research, and undertake high-level medical education. These ICUs operate 24 h a day, 7 days a week, and are staffed by specialized multidisciplinary teams including intensivists, critical care nurses, respiratory therapists, and other allied health professionals. This setting is chosen due to its relevance in providing critical care services, including the implementation of awake prone positioning for patients with severe respiratory conditions, such as those caused by COVID-19. The selection of multiple hospitals and ICUs allows for a broader understanding of nursing practices and experiences across different clinical environments within the region.

### Study population and sampling

The study population comprises registered critical care nurses working in the intensive care units of four Grade A tertiary hospitals in Hunan Province, China, where APP was routinely implemented. These nurses were directly involved in the care of patients undergoing APP. Inclusion criteria were registered critical care nurses, who held specialized critical care qualifications and were employed in intensive care units, with a minimum of one year of experience in caring for patients undergoing APP and willing to provide informed consent to participate in the study. Exclusion criteria were: (1) nurses who were on extended leave (e.g., maternity or sick leave) during the entire data collection period, and (2) nurses with less than one year of work experience in the ICU, regardless of their APP experience. The study employed purposive sampling to select participants who can provide in-depth and relevant insights into the research topic. The sample size was determined based on the principle of data saturation, where sampling continues until no new information is obtained, and further interviews do not yield additional insights. This approach ensured comprehensive coverage of the experiences and perspectives of critical care nurses involved in awake prone positioning, capturing a wide range of challenges, strategies, and emotional impacts associated with this practice. Data saturation is a key indicator of an adequate sample size in qualitative research, ensuring that the collected data sufficiently represents the study population’s experiences and viewpoints [16].

### Data collection

Data collection for this study was conducted through one-on-one semi-structured interviews in Mandarin Chinese. The interview guide was developed based on a thorough review of the literature and discussions within the research team. A pilot study involving three preliminary interviews was conducted solely to refine the interview questions; data from these pilot interviews were not included in the final analysis. The specific content of the interview guide is detailed in Table 1. The specific content of the final interview guide is detailed in Table 1. Semi-structured interview guide. Data collection took place between 1 January 2024 and 31 January 2024.

The specific process of data collection involved conducting interviews by the first author, primarily in the ICU nurses’ break room and the head nurse’s office. These locations were chosen to ensure a quiet, uninterrupted environment conducive to open and honest communication. Before each interview, participants were briefed on the study’s objectives, confidentiality measures, and other relevant details. To ensure confidentiality, all audio recordings and transcripts were de-identified using unique codes, stored on a password-protected computer, and will be destroyed after 5 years. Any potentially identifying details in quotes were altered or removed. General demographic data such as age, gender, educational background, and years of experience working in the ICU were collected.

Each interview lasted between 30 and 45 min and was recorded with the participant’s consent. Given the qualitative nature of this study, the sample size was determined based on the principle of data saturation, interviewing continued until no new information was obtained [17]. Data saturation was achieved after interviewing 16 critical care nurses.

**Table 1:** Semi-structured interview guide

(a) Can you describe your role in the practice of awake prone positioning?
(b) What difficulties did you encounter while nursing patients in the awake prone position?
(c) How do you collaborate with other colleagues while nursing patients in the awake prone position?
(d) How do you provide psychological support to patients undergoing awake prone positioning?
(e) What areas of training do you believe require further development or enhancement?
(f) What burdens do you experience when caring for patients undergoing awake prone positioning?
(g) Is there anything else you would like to add regarding your experiences in caring for patients undergoing awake prone positioning?

Note: Table 1 provides the interview guide used to explore critical care nurses’ experiences with awake prone positioning. This guide was developed specifically for this study and has not been published previously

### Data analysis

After completing the interviews, within 24 h, two researchers undertook the transcription of the recorded audio and notes. Following the transcription, a process of cross-verification was conducted between the researchers to ensure the accuracy of the transcribed data. This step involved comparing the transcribed text against the original audio recordings and notes, making any necessary corrections or clarifications. Once transcription and verification were complete, the transcripts were returned to the participants. This step allowed interviewees to clarify any ambiguous content, ensuring that their perspectives and experiences were accurately represented.

The study employed content analysis [18] to organize and analyze the transcribed material. Two researchers (H.S. and T.Z.) independently coded the data using NVivo 11, identifying themes and subthemes. This independent analysis helped mitigate bias and ensured a comprehensive understanding of the data. Following the initial independent coding, the research team convened to discuss the findings, reconcile different interpretations, and reach a consensus on the identified themes and sub-themes. Such collaborative analysis enhanced the analytical depth and validity of the findings. Finally, the refined themes were presented back to a subset of participants for confirmation (member checking), which further increased the credibility and trustworthiness of the research findings [19].

Throughout the analysis process, to enhance reflexivity, the research team regularly discussed how their clinical backgrounds might influence data interpretation. Reflective notes were maintained throughout data collection and analysis to ensure findings remained grounded in participants’ narratives rather than the researcher’s assumptions.

### Ethical considerations

Ethical approval was obtained from the Biomedical Research Ethics Committee of Hunan Normal University (IRB Approval Number: 2023316). Additionally, permission to conduct the study was obtained from the nursing administration and ICU directors of all four participating hospitals prior to data collection. Informed consent was obtained from all participants after they received a detailed explanation of the study. To protect participants, all interviews were conducted in private rooms (e.g., a nurses’ break room) with confidentiality assured. To ensure anonymity, all data were de-identified using unique codes immediately after collection; any potentially identifiable information was removed from transcripts and reports. Participation was voluntary, and participants could withdraw at any time without consequence.

## Findings

Sixteen critical care nurses participated in this study. There were eleven females (68.8%) and five males (31.2%) of the participants. The mean age was 32.6 years (range: 24–43 years), and the mean level of ICU work experience was 9.6 years (range: 2–24 years). The majority hold a Bachelor of Science in Nursing degree (*n* = 13, 81.2%) (Table 2).

**Table 2:** Participant demographic characteristics

Characteristic	Nurses (*N* = 16)
**Gender**	
Female	11 (68.8%)
Male	5 (31.2%)
**Age (years)**	
Mean ± SD (range)	32.6 ± 5.84 (24–43)
**Educational background**	
BSN	13 (81.2%)
MSN or above	3 (18.8%)
**Years of ICU work experience**	
Mean ± SD (range)	10.9 ± 6.34 (2–24)
**Professional title**	
Primary title	4 (25%)
Medium-grade professional title	10 (62.5)
Senior professional title	2 (12.5%)

Abbreviations: BSN, Bachelor of Science in Nursing; MSN, Master of Science Degree in Nursing

We conducted a thorough analysis of data collected from critical care nurses regarding their experiences with awake-prone positioning. The analysis revealed six main themes and thirteen corresponding sub-themes, which are summarized in Table 3. The quotations below are intended to maximally restore the original narratives of the critical care nurses. They have been contextualized, interpreted, and integrated into the text to provide a comprehensive understanding of their experiences with awake prone positioning.

**Table 3:** Themes and sub-themes

Themes	Sub-themes
The role of nurses in the practice of awake prone positioning	Patient assessment and preparation
	Technical execution and monitoring
Technical challenges in awake prone positioning	Difficulties in equipment usage
	Complexity of technical operations
	Diverse patient needs
Team collaboration and communication	Collaboration among nurses
	Interdisciplinary collaboration
Patient responses and psychological support provided by nurses	Patient responses
	Psychological support provided by nurses
Training and educational needs	Assessment of current training adequacy
	Required training directions
Nurses’ burden	Physical burden
	Emotional and Psychological Burden

Table 3 presents the main themes and sub-themes identified from the qualitative analysis of critical care nurses’ experiences with awake prone positioning

### The role of nurses in the practice of awake prone positioning

This theme illuminates the multifaceted and pivotal roles that critical care nurses assume in the management and implementation of APP. Beyond mere technical execution, nurses were involved from the initial planning stages through to ongoing patient management. The findings reveal that their role encompasses comprehensive patient assessment, meticulous technical execution, and continuous monitoring, all of which demand advanced clinical judgment, adaptability, and vigilance.

#### Patient assessment and preparation

Critical care nurses emphasized that thorough patient assessment and preparation were foundational to the safe and effective implementation of APP. This preparatory phase was described as a multi-dimensional process encompassing clinical evaluation, patient education, and physical preparation.

Clinical evaluation was identified as the critical first step to determine patient suitability and anticipate potential complications. As one nurse explained:

*Nurses conduct a thorough evaluation of the patient’s medical history*,* current health status*,* and specific needs. This includes assessing lung function*,* oxygenation levels*,* and potential contraindications for prone positioning. (RN3)*

Patient education was considered equally essential to foster cooperation and alleviate anxiety. Nurses recognized that informed patients were more likely to tolerate the discomfort associated with prolonged prone positioning. One participant described the educational approach: *“It is essential to inform the patient about the purpose*,* process*,* and potential benefits and risks of awake prone positioning. Nurses explain the procedure in understandable terms*,* ensuring the patient knows what to expect.” (RN16)*

Beyond explaining the procedure itself, nurses also focused on managing expectations regarding the logistics of care. Clear communication about treatment duration and goals helped align patient expectations with clinical realities: *“Clear communication about the duration of each prone session*,* the frequency of position changes*,* and the overall treatment goals helps set realistic expectations for the patient.” (RN9)*

Finally, physical preparation was undertaken to prevent procedure-related complications. Nurses conducted skin assessments and applied protective padding to reduce the risk of pressure injuries, a common concern with prolonged prone positioning. One nurse detailed this aspect: *“Physical preparation includes ensuring the patient is in a suitable physical condition for the positioning*,* such as checking for any skin integrity issues that could be exacerbated by prone positioning*,* and preparing the skin and protective padding as needed to reduce the risk of pressure sores.” (RN12)*

Collectively, these preparatory steps were viewed by nurses as essential investments in patient safety and procedural success.

#### Technical execution and monitoring

The technical execution of the APP and subsequent patient monitoring represented the core of nurses’ hands-on responsibilities. Participants described this phase as requiring both technical precision and continuous clinical vigilance.

Nurses emphasized the meticulous nature of positioning, where attention to even minor details could prevent serious complications.

*Every detail matters*,* from how we position the arms to avoid nerve damage*,* to monitoring pressure points to prevent skin breakdown. It’s a meticulous process that requires constant vigilance.* (RN12)

Beyond the initial positioning, nurses described the need for ongoing dynamic monitoring. Patient responses to the prone position were not static, requiring continuous assessment and real-time adjustments to care. One nurse captured this iterative process:

*We continuously monitor the patient’s vital signs*,* oxygenation levels*,* and comfort. Adjustments are often needed based on the patient’s response to prone positioning. It’s a dynamic process that requires us to be alert and responsive.* (RN15)

This combination of precise technical execution and responsive monitoring underscores the advanced clinical skills required of critical care nurses managing APP. Their ability to simultaneously attend to physiological parameters, patient comfort, and potential complications was central to the safe and effective delivery of this intervention.

### Technical challenges in awake prone positioning

Critical care nurses encountered multiple technical challenges when implementing APP, spanning equipment difficulties, procedural complexity, and the need to accommodate diverse patient populations. These challenges demanded not only technical proficiency but also adaptability and problem-solving skills.

#### Difficulties in equipment usage

Equipment-related challenges were frequently cited, particularly concerning the setup and operation of respiratory support devices. Nurses described struggling with complex equipment configurations, troubleshooting technical malfunctions, and adapting to varying device models across different clinical settings. One nurse elaborated:

*Dealing with equipment setup challenges in awake prone positioning can be demanding. Ensuring the precise assembly*,* configuration*,* and calibration of equipment is crucial*,* which includes high-flow humidified oxygen therapy machines and non-invasive ventilators. Challenges may arise in navigating complex setups*,* troubleshooting sensor errors*,* and adapting to variations in equipment models.* (RN1)

The diversity of equipment models further compounded these difficulties, as nurses were expected to maintain proficiency across multiple device types. Another participant noted: *“There are many types of equipment models*,* and it’s hard for nurses to adapt to all of them.”* (RN3)

#### Complexity of technical operations

Beyond equipment issues, nurses emphasized the inherent complexity of the APP procedure itself. The process was described as multi-step and demanding, requiring coordination of numerous clinical activities simultaneously:

*The steps involved in APP include assessment*,* patient preparation*,* team collaboration*,* positioning*,* monitoring*,* ongoing care*,* documentation*,* communication with interdisciplinary team*,* and patient education. Each step isn’t easy.* (RN10)

This complexity necessitated meticulous attention to detail and a high level of technical proficiency. As one nurse explained, successful execution required navigating intricate procedures with precision:

*Managing the complexity of technical operations in APP requires meticulous attention to detail. Navigating intricate procedures and ensuring precise execution demand a high level of technical proficiency.* (RN3)

#### Diverse patient needs

The technical challenges of APP were further amplified when caring for patients with unique or complex conditions. Nurses described needing to adapt standard procedures to accommodate individual patient characteristics, such as body habitus or physiological status:

*Prone positioning is particularly challenging for awake pregnant women and overweight patients. Meeting diverse patient needs during APP involves tailoring technical approaches.* (RN15)

This required nurses to move beyond rigid protocols and employ personalized strategies based on ongoing assessment of individual patient conditions (RN3). The ability to adapt technical approaches to diverse patient needs was thus identified as a critical competency for nurses managing APP.

### Team collaboration and communication

Effective teamwork and communication emerged as essential facilitators of successful APP implementation. Nurses described collaboration occurring at two levels: within the nursing team and across interdisciplinary boundaries. Both were viewed as critical to ensuring coordinated, safe, and holistic patient care.

#### Collaboration among nurses

Within the nursing cohort, seamless coordination was described as a cornerstone of effective APP delivery. Nurses emphasized that sharing insights and maintaining clear communication enabled precise execution of each procedural step. This intra-team collaboration was seen as enhancing care quality while reducing the likelihood of errors. One nurse captured this dynamic:

*Collaboration among nurses is our strength. We coordinate seamlessly during awake prone positioning*,* ensuring each step is executed with precision. Communication is key*,* and we share insights and observations to provide comprehensive care.* (RN11)

This collective approach allowed nursing teams to pool their expertise and maintain consistent vigilance throughout the procedure.

#### Interdisciplinary collaboration

Beyond the nursing team, collaboration with other healthcare professionals was equally valued. Nurses worked closely with physicians, respiratory therapists, and other disciplines to develop comprehensive care plans tailored to each patient’s needs. This interdisciplinary integration brought diverse perspectives together, enriching clinical decision-making and enhancing patient outcomes. As one participant explained:

*Collaborating with different disciplines brings a wealth of expertise to the table. As a nurse*,* working alongside physicians and respiratory therapists means we can integrate diverse perspectives*,* leading to well-rounded and effective care plans for our patients.* (RN6)

Such interdisciplinary teamwork was described as essential for addressing the multifaceted challenges inherent in APP, where respiratory management, positioning logistics, and patient comfort must be simultaneously optimized.

### Patient responses and psychological support provided by nurses

Nurses described attending not only to patients’ physiological needs during APP but also to their emotional and psychological well-being. This theme captures both the observed emotional responses of patients and the supportive strategies nurses employed to address them.

#### Patient responses

Participants observed that patients undergoing APP exhibited a range of emotional responses, largely related to the discomfort and unfamiliarity of the procedure. While some patients adapted smoothly, others experienced significant psychological distress. One nurse noted the common emotional reactions:

*Patients’ responses may include discomfort with the new position*,* feelings of anxiety*,* fear*,* or uncertainty.* (RN2)

The variability in patient adaptation was also highlighted, with individual differences shaping how patients experienced the procedure:

*Patient responses vary; some adapt smoothly to awake prone position*,* while others express initial discomfort.* (RN8)

These observations underscored the need for individualized psychological support tailored to each patient’s emotional state.

#### Psychological support provided by nurses

In response to these emotional needs, nurses described providing active psychological support as an integral component of their APP care. This support took multiple forms, including emotional comfort, validation of patient concerns, and information sharing to alleviate anxiety. One participant articulated this aspect of their role:

*Providing emotional comfort is a crucial part of our role. We listen*,* validate concerns*,* and offer reassurance*,* ensuring patients feel secure and understood throughout the awake prone positioning. It’s an integral aspect of holistic care.* (RN9)

Beyond immediate comfort, nurses emphasized the importance of building trusting relationships with patients over time. Such trust was seen as foundational to patient engagement and cooperation during the procedure:

*We work to create an environment where patients feel safe and understood during APP. When trust is established*,* it paves the way for effective collaboration and patient engagement.* (RN11)

This relational work, listening, validating, reassuring, and building trust, was described by nurses as essential not only for patient comfort but also for the successful completion of the procedure itself.

### Training and educational needs

Nurses consistently highlighted the importance of specialized training to meet the demands of APP care. They described gaps in current preparation and identified specific directions for future educational initiatives to enhance their competence and confidence.

#### Assessment of current training adequacy

Many participants expressed that existing training was insufficient to prepare them for the complexities of APP. While theoretical knowledge was often provided, nurses reported a lack of practical, hands-on experience that left them feeling unprepared for real-world application. One nurse stated bluntly:

*The current training is insufficient. I want to master the specific techniques more proficiently for positioning patients in the awake prone posture.* (RN4)

Another participant elaborated on the theory-practice gap, noting that without adequate simulation or supervised practice, transitioning from classroom learning to bedside execution remained challenging (RN7).

#### Required training directions

In response to these perceived gaps, nurses articulated clear priorities for future training. Three key areas emerged: hands-on practice, complication management, and psychological support skills.

Hands-on practice was identified as essential for building confidence and procedural proficiency. Nurses advocated for simulation-based learning that mirrored real clinical scenarios:

*We need more simulation-based learning and hands-on practice sessions that allow us to gain confidence and proficiency in performing awake prone positioning safely and effectively.* (RN5)

Complication management represented another critical training need. Nurses sought education on identifying, preventing, and managing potential APP-related complications, including pressure ulcers and airway issues:

*I think training that specifically addresses the identification*,* prevention*,* and management of potential complications associated with awake prone positioning*,* such as pressure ulcers*,* airway issues*,* or discomfort*,* is deemed necessary.* (RN8)

Finally, psychological support techniques were emphasized as an often-overlooked but essential component of APP training. Given the stress and discomfort patients frequently experience, nurses recognized the need to develop skills in emotional support and therapeutic communication:

*Given the stress and discomfort patients may experience during awake prone positioning*,* nurses have identified a need for training on how to provide emotional support and effectively communicate with patients to alleviate their anxiety and enhance their cooperation.* (RN11)

Collectively, these training priorities reflect nurses’ desire for a more comprehensive, practice-oriented educational approach that addresses both the technical and interpersonal dimensions of APP care.

### Nurses’ burden

Caring for patients undergoing APP imposed significant burdens on nurses, spanning physical exertion and emotional strain. These challenges arose from the procedure’s complexity, the sustained vigilance it demanded, and the psychological toll of supporting distressed patients in a high-stakes environment.

#### Physical burden

Nurses described the physical demands of APP as substantial, particularly during patient positioning. Maneuvering patients into the prone position required strength and precision, with the difficulty amplified when caring for obese or less mobile individuals. One participant highlighted the physical toll:

*The process of turning patients into the prone position requires a lot of physical effort*,* especially for obese patients or those with limited mobility. It’s physically demanding and can lead to muscle strain or fatigue.* (RN11)

The cumulative effect of this physical labor was evident in nurses’ descriptions of post-shift exhaustion. Another nurse shared the lasting impact:

*After a day of assisting with prone positioning*,* I often feel physically drained*,* and I feel a particular pain in my lower back*,* which can impact my energy levels and my ability to provide care in other aspects.* (RN13)

These accounts underscore the ergonomic challenges inherent in APP and their potential consequences for nurses’ physical well-being and sustained clinical performance.

#### Emotional and psychological burden

Beyond physical demands, nurses carried a profound emotional burden. They were tasked with providing continuous reassurance to vulnerable, anxious patients while managing their own emotional responses to witnessing distress. The empathetic connection nurses developed with patients made this aspect of care particularly taxing:

*Every shift*,* I witness the discomfort and anxiety of patients in prone positioning. It’s challenging to constantly see people in distress and not always be able to alleviate their fear immediately.* (RN13)

The uncertainty inherent in critical care further compounded this psychological strain. Despite recognizing the therapeutic potential of APP, nurses remained acutely aware that patient outcomes could be unpredictable:

*Working in critical care*,* you’re always on edge about patient outcomes. With prone positioning*,* even though we know it can help*,* there’s still uncertainty*,* and that uncertainty is stressful.* (RN16)

This combination of sustained empathy, exposure to patient suffering, and clinical uncertainty created a significant emotional load that nurses carried alongside their technical responsibilities.

## Discussion

This qualitative study delves into the nuanced experiences of critical care nurses caring for patients undergoing awake-prone positioning. The discussion encompasses the multifaceted challenges, emotional dimensions, and the invaluable insights gained through the narratives of the nursing cohort.

The core theme of intense workloads emerges as a pivotal aspect of awake prone positioning care. Nurses grapple with the intricacies of managing various catheters, monitoring equipment, and executing precise procedures, which is consistent with the findings of Hochberg CH [20]. The study underscores the demand for constant vigilance and quick decision-making, emphasizing the potential risks associated with catheter dislodgement, particularly during the delicate process of transitioning patients into the prone position.In addressing these challenges, there is a pressing need for systemic changes. Technological advancements, streamlined protocols, and a robust team collaboration framework can collectively contribute to mitigating the impact of intense workloads [21]. For example, implementing adjustable beds designed specifically for prone positioning can improve safety and reduce the physical burden on nurses. Streamlined protocols, such as standardized APP checklists, can ensure consistency across teams and improve interdisciplinary collaboration.

Moreover, ongoing training programs should be tailored to enhance technical proficiency and situational awareness [22], empowering nurses to navigate the complexities inherent in awake prone positioning.

Our findings emphasize the necessity for specialized training programs tailored to the intricacies of awake prone positioning care. From mastering patient positioning techniques to efficiently handling diverse catheters and equipment, nurses require targeted education to excel in their roles. Simulation-based training emerges as a valuable tool for honing practical skills and fostering confidence in high-pressure scenarios [23, 24].Institutions must recognize the dynamic nature of critical care and invest in continuous, comprehensive training initiatives [25]. These programs should encompass technical proficiency, effective communication strategies, and psychological resilience. By prioritizing specialized training, healthcare organizations can fortify their nursing teams to deliver optimal care in the challenging landscape of awake prone positioning.

The study illuminates the profound emotional toll that critical care nurses bear while tending to patients undergoing awake prone positioning, And this point is also mentioned in Morley G ‘s research [26]. The uncertainty surrounding patients’ conditions and the emotional distress they endure create a unique set of challenges [27, 28]. The findings highlight the importance of acknowledging and addressing the emotional burden faced by nurses as an integral part of providing holistic care.To foster emotional well-being, interventions should extend beyond traditional clinical approaches. Integrating psychological support services, peer mentoring programs, and creating spaces for debriefing and reflection are crucial steps toward building resilience in the nursing workforce [29]. By acknowledging the emotional dimensions, healthcare institutions can create a more compassionate and supportive care environment [30].

In awake prone positioning, multidisciplinary collaboration plays a pivotal role in ensuring the success and safety of the procedure. Effective multidisciplinary collaboration involves close coordination and communication between nurses, physicians, respiratory therapists, and other members of the healthcare team [31]. Each member brings unique expertise and perspectives to the table, contributing to a holistic approach to patient care. Nurses play a central role in facilitating multidisciplinary collaboration during awake prone positioning [15]. By implementing some possibly effective strategies, including sharing knowledge and experience, clarifying roles and responsibilities, establishing common treatment goals, interdisciplinary collaboration in awake prone positioning can be effectively promoted, leading to the delivery of more comprehensive and efficient patient care services. To operationalize effective communication and shared goals, SBAR [32] (Situation-Background-Assessment-Recommendation) framework can be used to standardize communication between team members. The model may provide structured pathways for improving collaboration, ultimately leading to better patient outcomes in APP.

This study contributes to the literature by providing in-depth, qualitative exploration of critical care nurses’ experiences with APP. Beyond confirming the technical challenges documented in previous research, it reveals the often-invisible dimensions of APP care, including the emotional labor of supporting conscious patients, the critical role of interdisciplinary teamwork, and the specific training gaps that nurses themselves identify. These insights move the discourse beyond clinical efficacy to highlight the human factors that are essential for sustainable, high-quality APP implementation.

### Implication

This study provides valuable insights into the experiences of critical care nurses caring for patients undergoing APP, with several key implications for clinical practice, education, and policy. In clinical practice, hospitals should establish standardized protocols for APP focusing on patient assessment, equipment use, and multidisciplinary teamwork. These protocols should be developed collaboratively by multidisciplinary committees involving nurse leaders, intensivists, and respiratory therapists, as they provide nurses with clear guidance and reduce decision-making uncertainty during complex procedures. In nursing education, both newly recruited ICU nurses and those pursuing critical care specialization need targeted training on APP, with particular emphasis on technical skills, patient positioning, and psychological support. Simulation-based training can be particularly helpful in building nurses’ confidence and clinical competence. Regarding nurses’ well-being, the physical and emotional burdens associated with APP highlight the need for better workload management and support systems. Hospitals should implement practical measures such as providing patient-lifting equipment, ensuring equitable workload allocation, and offering accessible mental health support, including peer support groups and counseling services. At the institutional level, hospitals should invest in ongoing education and foster teamwork among healthcare professionals. Developing detailed clinical guidelines and standard operating procedures for APP, overseen by nursing leadership in collaboration with intensive care units, is essential to ensure consistent, evidence-based practice. By implementing these recommendations, hospitals can improve both patient outcomes and nurse resilience, thereby sustaining high-quality care in critical settings.

### Limitations

This study has several limitations. First, although data saturation was achieved, participants were recruited from seven intensive care units across four tertiary hospitals in Hunan Province, China. This geographic and institutional specificity may limit the transferability of findings to critical care nurses in other healthcare systems or resource-limited settings.

Second, the first author’s clinical background in critical care nursing may have influenced participant responses during interviews. To minimize this potential influence, we employed dual independent coding, team discussions, and member checking.

Third, this study focused exclusively on nurses’ perspectives and did not directly incorporate patients’ voices. Future research should include patient perspectives to provide a more holistic understanding of the nursing care process in APP.

## Conclusion

This qualitative study delving into the experiences of critical care nurses caring for patients undergoing awake prone position ventilation offers valuable insights that bear significance for both clinical practice and future research. It is evident that by recognizing and addressing the challenges faced by critical care nurses, we pave the way for enhanced patient care and the nurturing of resilience within the nursing workforce.

Additionally, the study highlights the importance of providing ongoing support and specialized training for nurses to manage the physical and emotional demands of APP. Institutions should implement structured protocols and offer access to mental health resources, ensuring nurses are well-prepared and supported in their roles.

Future research should build on these insights, exploring the impact of sociocultural factors, patient perspectives, and refining interventions to continually elevate the standards of care in awake prone position ventilation. Through these efforts, we can collectively contribute to a more effective and compassionate healthcare landscape.

## Supplementary Information

Below is the link to the electronic supplementary material.

Supplementary Material 1

## Data Availability

No datasets were generated or analysed during the current study.
