# Supplementary material for: The experiences of critical care nurses caring for patients undergoing awake prone positioning: a qualitative study
Source: BMC Nurs. 2026 Mar 11;25:373. doi: 10.1186/s12912-026-04507-0 (PMC13088580; doi:10.1186/s12912-026-04507-0)
Supplement: Supplementary file 1 — Supplementary Material 1 [file 12912_2026_4507_MOESM1_ESM.docx]

# Interview guide

1. Can you describe your role in the practice of awake prone positioning?
2. What difficulties did you encounter while nursing patients in the awake prone position?
3. How do you collaborate with other colleagues while nursing patients in the awake prone position?
4. How do you provide psychological support to patients undergoing awake prone positioning?
5. What areas of training do you believe require further development or enhancement?
6. What burdens do you experience when caring for patients undergoing awake prone positioning?
7. Is there anything else you would like to add regarding your experiences in caring for patients undergoing awake prone positioning?
